# The impact of landscape and prey on psyllophagous ladybird communities in a tropical environment

**DOI:** 10.1371/journal.pone.0320898

**Published:** 2025-04-11

**Authors:** Marine Baujeu, Laura Moquet, Frederic Chiroleu, Antoine Becker-Scarpitta, Bernard Reynaud

**Affiliations:** 1 UMR PV BMT, Université de La Réunion, Saint-Pierre, La Réunion, France; 2 UMR PV BMT, CIRAD, Saint-Pierre, La Réunion, France; University of Carthage, TUNISIA

## Abstract

This study examines the community composition and structure of psyllophagous ladybirds in a tropical environment, focusing on their interactions with psyllids as a food resource. It investigates the effects of prey availability and landscape composition on the structure of all ladybird species associated with psyllids and on the presence and abundance of species whose life cycles depend on psyllids. Sampling was conducted in Reunion island on two psyllid-infested plant species, *Leucaena leucocephala* and *Acacia heterophylla*. Ladybirds and psyllids were regularly collected during two years using a thermal aspirator, and visual inspection was conducted at eleven sites visited monthly. In this study, 16 ladybird species were identified, and only juveniles from *Coccinella septempunctata, Exochomus laeviusculus,* and *Olla v-nigrum* were frequently present, suggesting they can complete their biological cycle on psyllids. The structure of psyllophagous ladybird communities in a tropical environment is driven by the psyllid host plant and the monthly average temperature. When studied separately, food resources or landscape variables did not affect significantly the communities. The distribution of *Coccinella septempunctata* is limited to high elevations, where it is recognized as an aphid-eating species, mainly in its juvenile form. Conversely, at low elevation, we encountered juvenile individuals of the generalist species *Exochomus laeviusculus* and the specialist species *Olla v-nigrum*. The presence and abundance of the generalist was positively influenced by the landscape and the presence of the specialist positively by prey abundance only.

## Introduction

An ecological community is a group of species coexisting in the same habitat and interacting with one another around shared resources [1]. Understanding the dynamics of ecological communities is a cornerstone of ecology. Communities are influenced by a range of abiotic factors including climate [2–6], land type and cover [2,7], resource availability [8,9], and geographical characteristics [10]. These factors collectively shape community composition, structure, and function, providing insights into species interactions, biodiversity, and ecosystem dynamics [1,11].

Research on ladybird communities (Coccinellidae) has significantly contributed to our understanding of these dynamics and has tested various concepts in community ecology [12]. The Coccinellidae family encompasses about 6000 species across 360 genera [13,14]. While most ladybirds are predators of other arthropods [15,16], some species are phytophagous or mycophagous [17]. A distinctive behavior of predatory ladybirds is their tendency to aggregate around abundant food resources [18–23], which is important for understanding their community structure. Whether or not the species feed obligatorily on prey, this aggregation creates interaction dynamics within the community, where competition and predation collectively shape the organization of all species present.

In temperate regions, prey availability is the main factor explaining the composition of aphidophagous ladybird communities at the habitat scale [24–27]. According to Honek & Evans, 2012 [28] they rely on different prey types: (i) essential prey, crucial for reproduction and larval development; (ii) accepted prey, a secondary less favourable food resource; and (iii) alternative prey, which sustains survival in scarcity. Aphidophagous ladybird communities include mobile generalist adults and sedentary specialist larvae reliant on specific prey. Prey abundance affects the presence of adults, female oviposition choices, and larval abundance [29,30]. Smaller ladybird species can persist at lower prey densities, while larger species require higher abundances to sustain their populations [31]. Additionally, ladybird adults possess prey detection thresholds, responding only when prey density reaches a certain level, which influences their distribution and aggregation patterns within habitats [32].

Landscape characteristics, including diversity, fragmentation, and the availability of host plants, play a role in structuring aphidophagous ladybird communities. Diverse landscapes, which offer a range of prey and habitats, particularly benefit generalist species that can exploit various resources due to their dietary flexibility [33]. In contrast, landscape fragmentation creates barriers that restrict movement and limit habitat access, disproportionately affecting species with low dispersal capacities, often smaller in size [34,35]. Finally, the availability of host plants for larvae directly influences the distribution of specialist species whose life cycles are closely tied to psyllids. These host plants support communities by providing breeding sites and a stable food resource for juvenile stages [27].

Most studies on ladybird communities have focused on aphidophagous species in temperate continental agricultural systems [7,23,33,36–38] but there is limited understanding of psyllophagous species. Psyllids, while less common prey, are essential for some Coccinellini [15]. Giorgi et al. (2009) [39] identified only three of 62 ladybird species as having psyllids as essential prey. Some studies confirm psyllids as essential for certain species [40–43]. Most research on psyllophagous ladybirds has been observational or correlational [41,43]. Nevertheless, many studies have explored the introduction of ladybirds to control psyllid pests [44–53]. Psyllids are highly specific to host plants, with fewer than 25 species being significant agricultural pests [54,55]. They feed on phloem, xylem, and parenchyma [56,57], sometimes transmitting pathogens such as *Candidatus* Liberibacter, which causes citrus greening disease [58,59].

Our study investigates the composition of psyllid-feeding ladybird communities on Réunion Island in the southwest Indian Ocean. The coexistence of obligatory or occasionally psyllophagous and non-psyllophagous species within the same micro-habitat leads to distinct interaction dynamics, where competition among psyllid feeders and predation by non-feeders collectively shape the structure of the ladybird community.

We aim to: (i) identify all species interacting with psyllid-feeding ladybirds, whether they consume psyllids occasionally, as a primary resource, or play other ecological roles within psyllophagous communities; (ii) investigate how prey availability influences the structure of psyllophagous ladybird communities, depending on their traits of dietary specificity and body size and (iii) explore how landscape characteristics shape the structure of psyllophagous ladybird communities, with a focus on how dietary specificity and body size traits influence species’ responses

We propose the following hypotheses: (i) Psyllophagous ladybird communities are primarily composed of generalist adults, with a minority of specialist species, including larvae, that depend on psyllids as essential prey (ii) increased psyllid abundance is expected to enhance the density of psyllophagous ladybird populations, particularly for specialist species whose larvae and adults rely on psyllids as a primary resource (iii) Diverse landscapes will favor generalist species due to increased prey diversity, availability of host plants is expected to enhance specialist species and landscape fragmentation is expected to disadvantage smaller species due to their limited dispersal capabilities.

## Materials and methods

### Study context

In the 1980s, widespread infestations of the psyllid *Heteropsylla cubana* Crawford, 1914 (Hemiptera: Psyllidae), severely impacted *Leucaena leucocephala* (Lam.) de Wit in many regions, causing extensive defoliation and threatening the viability of Leucaena as a key resource in agroforestry and grazing systems [60–62]. In response, biological control programs were implemented globally, including in La Réunion, where predatory ladybirds such as *Olla v-nigrum* (Mulsant, 1866) and *Curinus coeruleus* (Mulsant, 1850) were introduced to manage psyllid populations [63,64]. Currently, the IUCN classifies this plant species as one of the world’s 100 most invasive exotic plants. It can be found in Reunion island at elevations of between 0 and 400 meters.

Today, a new psyllid invasion poses a similar threat to an endemic species from La Réunion, *Acacia heterophylla* Willd., which is experiencing significant ecological pressure from the invasive psyllid *Acizzia uncatoides* (Ferris & Klyver, 1932). Since its detection in La Réunion in 2006, *A. uncatoides* has caused severe damage to *Acacia heterophylla* resulting in increased tree mortality and ecosystem disruption [65]. This recurring challenge raises questions about the role of biological control in managing invasive psyllid populations on the island. Tamarind Forests extend from an elevation of 1400 to 2000 m [66,67].

By studying these two host plants, *Leucaena leucocephala* and *Acacia heterophylla*, this research bridges past and future biological control strategies, examining how historical interventions can inform current approaches to manage psyllid invasions and protect La Réunion’s native flora.

### Site selection

**Fig 1:**
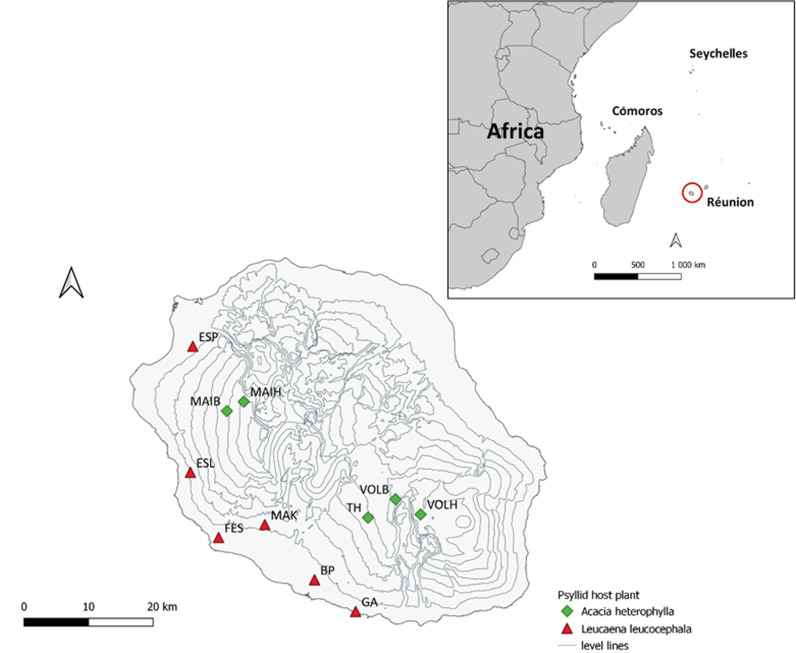
Locations of sampling sites on Reunion island, french island located at the south west of the Indien ocean. Elevation data from RGE ALTI® (source: IGN, 2023).

All sampling was carried out in Reunion island, a French oceanic island located in the south-western part of the Indian Ocean (long: 55°30’; lat: 21°11) (Fig 1). Reunion island is characterized by abundant rainfall, especially during the warm season. Rainfall distribution varies significantly across the island, with the south-eastern region receiving up to eight times more precipitation than the north-western region. Additionally, the island’s coastal and low-elevation areas typically experience lower levels of rainfall compared to its central, mountainous regions (Météo-France, 2020). Temperatures vary along the elevational gradient and across the seasons. At low elevations, on the coast, average temperatures vary between 20°C and 30°C during the wet season. In the dry season, temperatures can drop to 15°C at night. Temperatures are lower in mountainous regions and temperatures close to 0°C have been recorded at the highest peaks. Annual solar radiation intensity ranges from 1400–2500 h and can reach 2900 h at elevations below 400 m [68].

Coccinellidae sampling design has been based on the psyllid host plants’s presence, and we specifically targeted two heavily infested species: *Leucaena leucocephala* and *Acacia heterophylla*.

Eleven sites were sampled (Fig 1, source: [69]): six at low elevations corresponding to the distribution area of *L. leucocephala* and five at high elevations corresponding to the distribution area of *A. heterophylla.* The sites were chosen based on accessibility and the high availability of acacia and leucaena host plants within an approximate area of 50 m^2^ to facilitate sampling. All the sampling sites are located on the less humid west coast, which reduces the likelihood of frequent psyllid leaching.

### Psyllids and ladybird counts

Each site was sampled between 5 and 10 times (Table 1) between December 2020 and April 2022 during the hot rainy season. For each sampling (=each visit to a site on a given date), monthly average temperature, monthly cumulated precipitation and monthly average solar radiation intensity were calculated over the thirty days preceding the sampling using data from the Smart IS database (©CIRAD, 2022, https://smartis.re/).

**Table 1:** Site characteristics and sampling effort.

*Site name*	*Site identification*	*Altitude (meter)*	*Number of sampling*	*Number of trees sampled*
*Volcan Haut*	*VOLH*	2400	7	83
*Volcan Bas*	*VOLB*	1900	6	65
*Tamar’haut*	*TH*	1400	10	84
*Maïdo Haut*	*MAIH*	2000	5	70
*Maîdo Bas*	*MAIB*	1500	7	76
*Etang de Saint-Paul*	*ESP*	65	7	56
*Etang Saint-Leu*	*ESL*	380	6	57
*Foret de l’Etang Salé*	*FES*	65	10	87
*Les Makes*	*MAK*	400	6	50
*Bassin Plat*	*BP*	120	9	79
*Grande Anse*	*GA*	35	8	85
		*Totals*	*81*	*792*

A Garden Vacuum (Garden Vacuum STIHL® D-71336 Waiblingen), coupled with a fine mesh cloth, was used to collect the samples by vacuuming for one minute. Each sampling involved collecting one sample from each of ten unique trees, resulting in ten samples per sampling session. Using this method, we were able to quantify the psyllid population based on the presence of adult psyllids. Each sample was complemented with a hand collecting of the ladybird larvae and adults’ ladybirds found on the same branches previously sampled with the GVAC. This made ladybird sampling more effective and provided an accurate estimate of ladybird abundance and species richness.

We counted the ladybird larvae and adults, as well as adult psyllids. Ladybird larvae and adults were identified in the laboratory, using the keys provided by Chazeau et al. 1974 [70], supplemented by Quilici et al. 2003 [71], Poussereau et al. 2018 [72] and Magro et al. 2020 [73].

The pupal stage is immobile; therefore, the presence of pupae is directly linked to the presence of larvae. For our analyses, we added up the number of larvae and nymphs per tree to give us the number of “juveniles”.

We crosschecked our identifications for the juvenile ladybird stages. This involved randomly selecting 10% of the individuals for molecular identification by sequencing the traditional cytochrome oxidase 1 using LCO/HCO primers [74]. The sequences obtained were processed with Geneous® software v 10.2.6 (https://www.geneious.com) and compared with BLAST (Basic Local Alignment Search Tool), using the GenBank® reference database. The identifications were validated for all juveniles with a pair-wise sequence identity greater than 97%.

Psyllid adults were counted one by one when their number did not exceed 1000 individuals per sample. When samples had more than 1000 individuals, we separated the psyllid adults and juveniles using a filtration and sieving system (mesh size =  1 mm). The psyllid adults recovered in this way were freeze dried (ALPHA® 1-2 LDplus) for 24 hours and then weighed using a precision balance (Cubis® MCE125P-2S00-A). The number of psyllids was estimated by dividing the mass of psyllids by the average mass of one freeze-dried psyllid, previously determined from 1000 individuals (m_*H.Cubana*_ = 0.077 ±  0.012 mg; m_*A.uncatoides*_ = 0.111 ±  0.020 mg).

### Measures of landscape composition

We studied the landscape using QGIS® mapping software (version 3.22.7-Białowieża).

A geographical reference point was positioned at the centre of each site. The coordinates were read using a handheld GPS (Garmin Forerunner® 745). The land use map created by CIRAD [75] provided raw data (crop type, natural vegetation type, building type/inert surface). This allowed us to calculate the diversity of vegetation cover index for a radius of 1 km [23,33] around the geographical reference points using Simpson’s index, which considers habitat richness and landscape homogeneity. The equation for Simpson’s index is as follows:


$$D=\frac{1}{\sum \left(p_{i}\right)²}$$

pi is the proportion of habitat in the i^th^ land use category, based on 29 landscape categories (refer to Supplementary S1 Table. and S2 Fig. Data for details).

Using the same radii around the geographical reference points, we also counted the number of different landscape patches to determine landscape fragmentation.

### Statistical analysis

All data processing and statistical analysis were carried out using R (R Core Team, 2023, version 4.2.3).

For every statistical analysis conducted, with the exception for canonical multivariate analysis, we opted to analyze data from psyllid and ladybird sampling on *A. heterophylla* and *L. leucocephala* separately, mainly because of the significant difference between community composition and ecosystem structure. Additionally, individuals collected using both the GVAC method and manual collection from the same tree were combined for analysis.

#### Ladybird community structure.

For the community analysis, given the different numbers of trees sampled per survey, we averaged numbers of ladybird juveniles and adults per species and the psyllid abundance per survey -i.e., the numbers of individual collected using the thermal aspirator. To preserve the quality of the information, species abundance data were transformed into the Hellinger distance matrix following the approach presented byLegendre et al. (2001)[76]. Environmental variables were standardized.

To test the relationships between ladybird community composition and the landscape (i.e., Simpson’s Landscape Diversity Index, fragmentation and psyllid host plant cover), the climate (i.e., the monthly average temperature, rainfall and solar intensity radiation over the thirty days prior to sampling) and the prey abundance we performed a canonical analysis using a redundancy analysis, with the function *rda* from the R package ‘vegan’ v2.6.4 [77]. The RDA including ladybird communities associated with *Acacia heterophylla* and *Leucaena leucocephala*. The global model (drived with all data) included the psyllid host plant (*A. heterophylla* or *L. leucocephala*) as an explanatory variable to account for its influence on community structure.

To separate the effect of site variables (Simpson’s Landscape Diversity Index, fragmentation and psyllid host plant cover) from that of the other explanatory variables on community composition, in order to control pseudo-replication effects, we build a partial RDA by setting all the parameters relating to the site as a condition factor in the model.

We conducted three independent models: one model with all data including the psyllid species as an unconstrained variable only for this model; two models with a subset of the data including only the community associated with only one host species: *A. heterophylla*; or *L. leucocephala*. All three models followed the same structure:

The final model followed this structure:


$$\begin{array}{l} rda(matrix\_community\sim \;monthly\_cumulated\_rainfall+\;monthly\_average\_temperature+\\ \;monthlyaverage\_solar\_radiation\_intensity+\;food\_resource+\;\left(hostplantspecies\right)+\\ \;Condition(Simpson’sLandscapeDiversityIndex+\;landscape-fragmentation+\;\\ psyllid\_host\_plant\_cover)) \end{array}$$

The models were tested using *anova* function with 999 permutations. The adjusted-R^2^ will quantify the variance explained by all environmental variables.

Beta diversity was analyzed separately using the betadisper function, which assesses the dispersion of species composition across sampling sites. This analysis compared the variability in species composition between communities associated with the two host plants, independent of the RDA model.

#### Presence and abundance of ladybirds *whose life cycle depends* on psyllids.

To assess the effect of landscape (Simpson’s Landscape Diversity Index, fragmentation and psyllid host plant cover), climate (monthly average temperature, monthly cumulated rainfall and monthly average solar intensity radiation over the thirty days prior to sampling), and prey resource (number of psyllids collected in one minute using the GVAC) on the presence and abundance of ladybird adults and juveniles whose life cycle depends on psyllids, we used a generalized linear mixed-effects model (GLMM) through the **‘**glmer**’** function from the ‘lme4’ R package v1.1.35 [78]. GLMM is well-suited for analysing complex datasets where the response variable does not follow a normal distribution and may exhibit non-constant variance, discrete outcomes or other non-Gaussian characteristics. Moreover, we collected our samples several times at the same sites, i.e., our data was not independent. Therefore, careful analysis was essential to avoid any criticism of an artificial increase in degrees of freedom (i.e., so-called pseudo-replication) [79]. Pseudo-replication refers to the incorrect analysis or interpretation of replications that are not statistically independent, such as repeated observation of the same subjects (in our case, populations) [80]. However, GLMMs (generalized linear mixed models) are capable of handling hierarchically structured and unbalanced datasets, similar to ours, and effectively solving the statistical problem of pseudo-replication [79,81]. Specifically, we included random intercepts for both the site identifier and the sampling event. By incorporating random effects, we enhance the robustness of our statistical model, by accounting for the unique characteristics associated with each site and each sampling event in our study. This comprehensive framework provides a more accurate representation of the underlying data structure and strengthens the validity of our statistical inferences.

Therefore, for this analysis, we chose to focus on just three species found in their juvenile stages on psyllid colonies: *Coccinella septempunctata* Linnaeus, 1758 on *A. heterophylla*; and *Exochomus laeviusculus* Weise, 1909 and *Olla v-nigrum* (Mulsant, 1866) on *L. leucocephala*. Given the wide range of values, we log-transformed the number of psyllids collected per minute to help models convergence. Presence/absence was analysed for each development stage (adult and juveniles), using nested GLMER assuming binomial distribution with species background and landscape, climatic and food resource variables as fixed factors, the statement as a nesting factor, and site sampling as a random factor. The abundance of adults and juveniles for the 3 species were analysed by nested GLMER with Poisson distribution and the same explanatory variables. The significance of each specified fixed effect in the model was assessed using “Type II Tests of Fixed Effects”. We checked the “normality” of residuals, homogeneity of variance and independence of residuals.

## Results

### Global ladybird community structure

**Fig 2:**
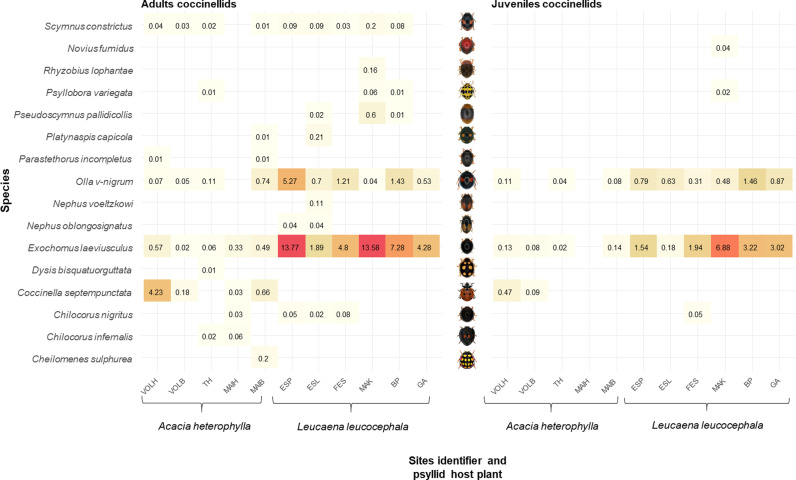
Heatmap showing the mean number of individuals per tree sampled (abundance) for each ladybird species, sites and psyllid host plant, for adults (left) and juveniles (right). The colour gradient from red to beige indicates a low to high relative abundance. **Images provided by Antoine Franck, CIRAD.**

A total of 4259 adult and 1539 juvenile ladybirds were collected. Sixteen species were identified, including 15 at the adult stage and 6 at the larval stage (Fig 2).

Across the study design, *Exochomus laeviusculus, Olla v-nigrum* and *Coccinella septempunctata* were the three most abundant species.

**Fig 3:**
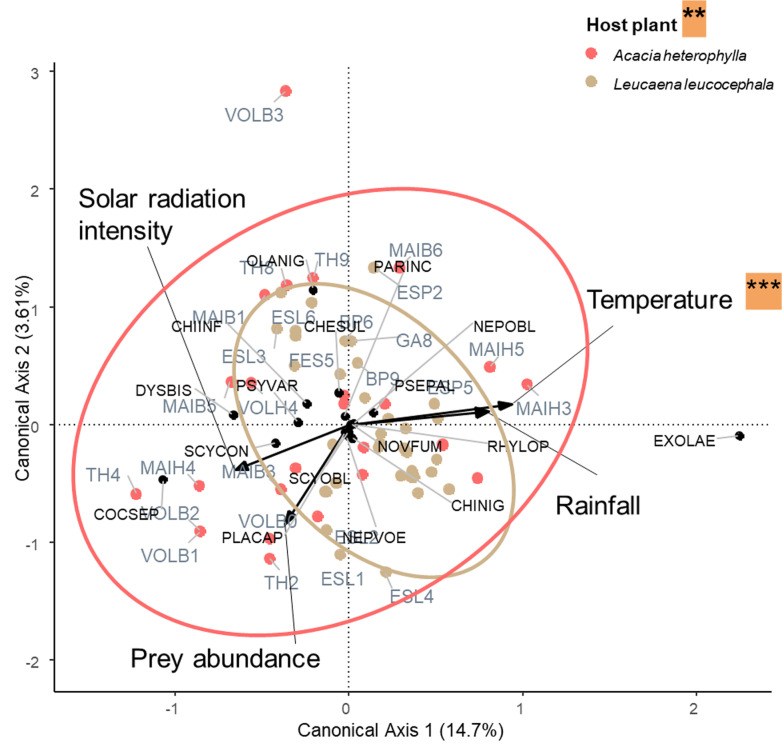
Canonical analysis including all data (first models), testing the relationships between sites (in blue and red), ladybird species (black points), and environmental variables (arrows). Ellipses show 95% confidence limits for each plant host group: *Acacia heterophylla* (red points) and *Leucaena leucocephala* (green points).

The beta diversity of the two sub-communities is significantly different, with a larger difference in composition for sites associated with *Acacia heterophylla* compared to *Leucaena leucocephala* (average distance to median group *Acacia heterophylla* =  0.634; group *Leucaena leucocephala* =  0.262, betadiper test: F-value =  41.676, Df =  1, P-value < 0.001). This result is supported by Fig 3, showing that the sub-community of psyllid ladybirds generally found at high elevations associated with *A. heterophylla* (in red in the Fig 3) has a broader ellipse compared to the sub-community of psyllid ladybirds generally found at low elevations associated with *L. leucocephala* (in brown in the Fig 3).

Species codes: Ladybirds: CHESUL: *Cheilomenes sulphurea*; CHIINF: *Chilocorus infernalis*; CHINIG: *Chilocorus nigritus*; COCSEP: *Coccinella septempunctata*; DYSBIS: *Dysis bisquatuorguttata*; EXOLAE: *Exochomus laeviusculus*; NEPOBL: *Nephus oblongosignatus*; NEPVOE: *Nephus voeltzkowi*; OLANIG: *Olla v-nigrum*; PARINC: *Parastethorus incompletus*; PLACAP: *Platynaspis capicola*; PSEPAL: *Pseudoscymnus pallidicollis*; PSYVAR: *Psyllobora variegata*; RHYLOP: *Rhyzobius lophanthae*; NOVFUM: *Novius fumidus*; SCYCON: *Scymnus constrictus*; See Table 1 for site codes.

The first canonical analysis including all data was statistically significant (F-value =  2.71, Df =  7, P-value =  0.001) and explained 50.02% of the total variance. The conditioned variables linked to the site (i.e., *Simpson’s Landscape Diversity Index, fragmentation and psyllid_host_plant_cover*) explained 37.35% of the total variance, while the explanatory variables (i.e., monthly *average_temperature, cumulated rainfall and solar intensity_radiation and prey_abundance*) explained 12.07% of the total variance. In the general model, the monthly average temperature and the psyllid host plant cover significantly influence the structure of the entire psyllophagous ladybird community (F-value =  5.51, Df =  1, P-value =  0.001; F-value =  5.05, Df =  1, P-value =  0.004, respectively).

### Ladybird community structure in specific-host-plant

*Leucaena leucocephala -* We found eleven ladybird species associated with *Leucaena leucocephala*, with a total of 3,621 adults and 1,447 juveniles. The site ESL and MAK were the most diverse, with 8 and 6 species collected, respectively. The site ESP had the greatest abundance of ladybirds, with an average of 19.21 adults and 2.32 juveniles per tree in average, ahead of MAK, with an average of 14.64 adults and 7.42 juveniles per tree in average. The two most abundant species were *Exochomus laeviusculus* and *Olla v-nigrum*, with 2,915 and 600 individuals, respectively, i.e., 80.5% and 16.6% of the total individuals collected at all low elevation sites (Fig 2). Canonical analysis conducted on the ladybird community associated with *L. leucocephala* (cf second RDA model in section statistical analysis) is not statistically significant (F-value =  1.01, Df =  4, P-value =  0.422), and no variables emerge as a significant driver of ladybird’s abundance matrix.

*Acacia heterophylla* - We found eleven ladybird species *associated with Acacia heterophylla*, with a total of 638 adults and 92 juveniles. The sites TH and MAIB showed the highest richness, with six species recorded at each site (Fig. 2). In addition, VOLH exhibited the highest relative abundance of ladybirds per tree, with an average of 4.91 adults and 0.14 juveniles per tree, followed by MAIB, with 2.11 adults and 0.22 juveniles per tree. However, in terms of total abundance, *Coccinella septempunctata* was the most abundant species across all sites, with 415 adults and 45 juveniles collected. Canonical analysis conducted on the ladybird community associated with *Acacia heterophylla* is not statistically significant (F-value =  0.76, Df =  4, P-value =  0.70, and no variables emerge as a significant driver of the ladybird’s abundance matrix variance.

### Presence and abundance of species whose life cycle depends on psyllid

*Leucaena leucocephala -* Models show (Table 2A) that the presence of *E. laeviusculus* adults was negatively influenced by landscape fragmentation and by host plant cover and positively influenced by Simpson’s Landscape Diversity Index and prey abundance. The presence of *O. v-nigrum* adults and juveniles (Table 2A & B) was only influenced by prey abundance.

**Table 2:** The statistical significances of the General Linear Mixed Models for the presence/absence data for adults (A) and Juveniles (B) of each ladybird species which use psyllid as an essential food. We analized data from *Exochomus laeviusculus* and *Olla v-nigrum* collected on *Leucaena leucocephala* and data from *Coccinella septempunctata* collected on *Acacia heterophylla*. Significant values are shown in bold. The low data quantity of juveniles of *Coccinella septempunctata* did not permit the attainment of model convergence (N.D.). Statistical values codes: E: EstimateE; Z: Z-value; P > z: P-value.

(A) Adults										
Psyllid plant host		*Leucaena leucocephala*						*Acacia heterophylla*		
Variables		*Exochomus laeviusculus*			*Olla v-nigrum*			*Coccinella septempunctata*		
		*E*	*Z*	*P < z*	*E*	*Z*	*P < z*	*E*	*Z*	*P < z*
Landscape	Simpson’s Landscape Diversity Index	**9.73**	**2.34**	**0.01**	−1.72	−0.25	0.80	46.26	1.48	0.14
	Fragmentation	**−0.01**	**−2.96**	**0.003**	0.002	0.44	0.66	−0.002	−0.61	0.54
	Host plant covering	**−0.02**	**−2.23**	**0.03**	0.02	1.43	0.15	**0.12**	**0.05**	**0.02**
Climatic	Rainfall	−0.01	−1.81	0.07	0.004	1.35	0.18	−0.001	−1.09	0.27
	Temperature	0.27	1.02	0.31	−0.23	−1.02	0.31	−0.29	−1.71	0.09
	SRI	0.0001	0.10	0.92	−0.001	−1.09	0.28	−0.004	−1.81	0.07
Food ressource	Prey abundance	**0.66**	**6.84**	**<0.001**	**0.35**	**3.82**	**<0.001**	**0.45**	**5.74**	**<0.001**
**(B) Juveniles**										
Psyllid plant host		*Leucaena leucocephala*						*Acacia heterophylla*		
Variables		*Exochomus laeviusculus*			*Olla v-nigrum*			*Coccinella septempunctata*		
		***E***	***Z***	***P < z***	***E***	***Z***	***P < z***	***E***	***Z***	***P < z***
Landscape	Simpson’s Landscape Diversity Index	1.34	0.19	0.85	2.86	0.54	0.59	N.D.		
	Fragmentation	−0.003	−0.65	0.52	0.002	0.35	0.73			
	Host plant covering	−0.01	−0.34	0.74	−0.001	−0.06	0.95			
Climatic	Rainfall	−0.003	−1.44	0.15	−0.001	−0.22	0.83			
	Temperature	0.16	1.15	0.25	0.42	1.28	0.20			
	SRI	−0.001	−0.95	0.34	−0.001	−0.15	0.88			
Food resource	Prey abundance	**0.53**	**7.10**	**<0.001**	**0.47**	**4.15**	**<0.001**			

The abundance of *E. laeviusculus* adults (Table 3A) was negatively influenced by landscape fragmentation and by host plant cover, and positively influenced by landscape diversity and prey abundance, but not by climate. Abundance of *E. laeviusculus* juveniles (Table 3B) was only positively influenced by prey abundance. Abundance of *O. v-nigrum* adults and juvenils (Table 3A & B) was not influenced by parameters included in our models.

**Table 3:** The statistical significances of General Linear Mixed Models for adults (A) and Juveniles (B) abundance of each ladybird species which use psyllid as an essential food. We analized data from *Exochomus laeviusculus* and *Olla v-nigrum* collected on *Leucaena leucocephala* and data from *Coccinella septempunctata* collected on *Acacia heterophylla*. Significant values are shown in bold. The low data quantity of *Coccinella septempunctata* juveniles did not permit obtaining values for fragmentation and host plant covering variable (N.D.). Statistical values codes: E: EstimateE; Z: Z-value; P > z: P-value*.

(A) Adults										
Psyllid plant host		*Leucaena leucocephala*						*Acacia heterophylla*		
Parameters		*Exochomus laeviusculus*			*Olla v-nigrum*			*Coccinella septempunctata*		
		*E*	*Z*	*P < z*	*E*	*Z*	*P < z*	*E*	*Z*	*P < z*
Landscape	Simpson’s Landscape Diversity Index	**3.36**	**2.46**	**0.01**	−0.65	−0.30	0.77	−5.87	−0.49	0.64
	Fragmentation	**−0.004**	**−3.43**	**<0.001**	0.001	0.13	0.90	0.0003	0.102	0.92
	Host plant covering	**−0.01**	**−2.06**	**0.04**	−0.003	−0.61	0.54	−0.002	−0.01	0.92
Climatic	Rainfall	0.002	1.41	0.16	0.002	1.26	0.21	0.003	1.95	0.05
	Temperature	−0.09	−1.02	0.31	0.14	1.20	0.23	−0.05	−0.33	0.74
	SRI	0.000	0.96	0.34	−0.001	−1.37	0.17	0.001	1.65	0.10
Food resource	Prey abundance	**0.12**	**8.18**	**<0.001**	0.01	0.21	0.84	0.019	1.00	0.32
**(B) Juveniles**										
**Psyllid plant host**		***Leucaena leucocephala***						***Acacia heterophylla***		
**Parameters**		***Exochomus laeviusculus***			***Olla v-nigrum***			***Coccinella septempunctata***		
		***E***	***Z***	***P < z***	***E***	***Z***	***P < z***	***E***	***Z***	***P < z***
Landscape	Simpson’s Landscape Diversity Index	0.99	0.61	0.54	−0.68	−0.19	0.85	29.29	2.01	0.05
	Fragmentation	2.79	−0.87	0.38	0.004	1.12	0.27	N.D.		
	Host plant covering	−0.003	−0.36	0.72	−0.0001	−0.02	0.99			
Climatic	Rainfall	−0.004	−0.56	0.58	−0.002	−0.84	0.40	−0.001	−0.45	0.65
	Temperature	−0.001	0.68	0.50	0.34	1.75	0.08	1.63	3.31	0.001
	SRI	0.07	−2.34	0.02	-0.002	−1.63	0.10	−0.003	−1.27	0.21
Food resource	Prey abundance	**0.17**	**6.49**	**<0.001**	0.06	1.20	0.23	0.57	1.45	0.15

*Acacia heterophylla* - The low number of *Coccinella septempunctata* juveniles collected during our sampling did not allow us to test the influence of all the landscape-related parameters on their abundance and the presence/absence model did not converge. Thus, we performed the analysis on adults alone (Table 2A).

The presence of *Coccinella septempunctata* adults (Table 2A) was positively influenced by host plant cover and prey abundance. The effect of the weather (rainfall) on the abundance of *C. septempunctata* adults (Table 3A) was near to the significance threshold. For *C. septempunctata* juveniles (Table 3B), temperature had a significant positive effect on their abundance.

## Discussion

### Species richness

We identified 16 species of ladybirds associated with the psyllid resource, 15 species were found at the adult stage and 6 at the juvenile stage. Poussereau et al. 2018 described 26 ladybird species in Reunion island. This species richness increased after reports of *Coccinella septempunctata* in 2020 [82] and the description of *Nephus apolonia* by Magro et al. 2020. The species richness identified around psyllid resources represented about 55% of the total ladybird diversity on the island.

Only 3 of the 16 species were collected in large quantities (over 20 individuals). Thus, our community of psyllophagous ladybirds was mainly composed of rare species, with three predominant species. This follows a classic pattern found in community ecology [83], which also applies to ladybirds [27,84].

Six of these species are natives to Reunion island: *Scymnus constrictus* Mulsant, 1850, *Cheilomenes sulphurea* (Olivier, 1791)*, E. laeviusculus, Dysis bisquatuorguttata* Mulsant, 1850*, Platynaspis capicola* Crotch, 1874 and *Psyllobora variegata* (Fabricius, 1781). Other species were either introduced accidentally or, in most cases, deliberately for biological control programmes. Only *C. septempunctata* is native to temperate climates. Among the 15 adult species, four are mentioned as psyllophagous in the literature, namely *E. laeviusculus, D. bisquatuorguttata, P. capicola*, and *O. v-nigrum* [70–72]. While *E. laeviusculus* and *O. v-nigrum* were the most abundant species in our study, the rarity of *D. bisquatuorguttata* and *P. capicola* can be attributed to different factors. Chazeau (1974) [70] described *D. bisquatuorguttata* as an infrequent species reflecting its naturally low abundance. *P. capicola*, despite its noted tolerance to climatic variability [70], may face competition from the numerous exotic ladybird species that dominate the island’s ecosystems [85]. Investigating whether specific site characteristics, such as landscape [33] or prey availability, provide a competitive advantage to native species like *P. capicola* would be an interesting avenue for future research. It is likely that the other species are present because they feed on psyllids, which are accepted or alternative prey (e.g., *Chilocorus nigritus* (Fabricius, 1798) and *Novius fumidus* (Mulsant, 1850)). Other species may use the resources associated with psyllids or the host plant (sooty mould, honeydew, e.g., *P. variegata*) [28].

Lastly, six species were collected in the juvenile stage, which suggests that they complete their life cycle on psyllids, using this resource as an essential prey. Among them, one larva of *C. nigritus*, one of *N. fumidus*, both strictly coccidiphagous, and one larva of *P. variegate*a, strictly mycophagous, are clearly accidentale collections [71]. The other three species, *E. laeviusculus, O. v-nigrum*, and *C. septempunctata*, were found in sufficient abundance to rule out the hypothesis of accidental collections. The detection of predatory ladybird larvae alongside a specific prey species is typically viewed as reliable evidence for assessing prey specificity in a natural setting. However, caution is advised in habitats with multiple potential prey species.

*E. laeviusculus* is a generalist species that feeds on aphids, mealybugs or psyllids as essential prey. This species is relatively small, with an average size of 3.6 mm [72]. It is the smallest species relying on psyllids for its life cycle in our study. *Olla v-nigrum* is described in the literature as a specialist predator that eats aphids and psyllids [15]. This tropical ladybird is native to French Polynesia and was voluntarily introduced to Reunion island in 1990 as part of a biological programme to control the psyllid *H. cubana* [63]. *Olla v-nigrum* measures between 4.4 and 6.0mm [86], which makes it the medium-sized species whose life cycle depends on psyllids in our study.

Contrary to *E. laeviusculus and O. v-nigrum, C. septempunctata* is widely known as a continental aphidophagous species [87–89]. Originally from Eurasia, a temperate continental region, its presence in Reunion island was reported in March 2020. This species measures between 5.0 and 8.0 mm [90] and is the largest species whose life cycle depends on psyllids in our study. The climate in Reunion island is almost temperate at high elevations, therefore, it is not surprising that *C. septempunctata* is found at high elevations. However, given its food preference, its presence in adult and larval stages on the psyllid *A. uncatoides* populations was unexpected. Their unusual use of psyllids as essential prey could be explained by the lifting of dormancy, which may occur in Reunion island in October, with the increase in daylight hours and temperature [91]. It is possible that *C. septempunctata* is abundant on aphids at high elevations and some individuals make a host jump.*C. septempunctata* populations are often observed in psylla colonies on both wild and cultivated pistachio trees in the spring and autumn, which coincides with the periods when there is a decline in the aphid populations found on herbaceous plants [92,93]. Mills [94] also noted in 1981 that during the early and late seasons, adults may be observed in association with prey types and habitats not used for reproduction. Furthermore, the anatomy of *C. septempunctata* is not adapted to the shrub layer because it lacks an enlarged anal disc that would help prevent it from falling [95]. In its native habitat, *C. septempunctata* prefers herbaceous to shrubby strata [96]. This species is rarely found on trees, such as *A. heterophylla*. We only found *C. septempunctata* juveniles at volcanic sites (VOLH and VOLB), where *A. heterophylla* are smaller.

### Community structuration

We were able to demonstrate that the psyllophagous ladybird community was structured by the psyllid host plant and the monthly average temperature in a tropical island environment. We found 11 species associated with *A. heterophylla* and 11 with *L. leucocephala*. It was also far less abundant than the low elevation community, associated with *L. leucocephala* (730 individuals associated with *A. heterophylla* versus 5068 with *L. leucocephala*). The results are not surprising given the differences between the two sub-communities in terms of landscape, climate, host plants and psyllid species. The lack of predators at high elevations means that psyllid populations are not controlled [97,98], and annual outbreaks occur [65]. The area of distribution of the psyllid host plant *Leucaena leucocephala* extends from 0 to 400 m above sea level and that of *Acacia heterophylla* from an elevation of 1400 to 2000 m (Strasberg et al. 2005; Le Roux et al. 2014). Therefore, the two variables, psyllid host plant and monthly average temperature, are correlated. Moreover, psyllid species are strongly associated with plant species (*A. uncatoides* on *A. heterophylla* and *H. cubana* on *L. leucocephala*). We cannot clearly determine which of the three variables – monthly average temperature, psyllid host plant or psyllid species - has the most influence on ladybird community structure.

When studied separately, none of the studied factors related to landscape, climate or prey resources structure the two sub-communities. Camarota et al. [99] have shown that landscape structure has no significant influence on the distribution of insect species in tropical forests. However, it is widely demonstrated that landscape composition [7,33,38,100], climate [101,102], and prey availability are the primary factors that induce ladybird assemblages; adults seek prey not only for their own welfare, but as a resource for their progeny [24–27,29,100]. It is possible that in our tropical environment, the psyllid resource is not essential for ladybird communities because a large number of ladybird species feed on psyllids as an alternative or accepted prey. The non-significant result may also indicate that the variables considered in our analysis, which were selected on the basis of knowledge or hypotheses, may not be the main drivers of the community patterns observed. It would be interesting to study other factors that influence ladybird communities, for example, other psyllid species [103], agrochemical use [104–106], the invasion rate of non-native species [100,107–110], or interactions with other predators or parasites [111–116]. The temporal scale should also be considered [117] to improve our understanding of the fluctuations in the ladybird communities

### Species whose life cycle depends on psyllid

We demonstrate that the main area of distribution of the two tropical species, whose life cycle depends on the psyllids, *Exochomus laeviusculus* and *Olla v-nigrum*, is at lower elevations. The temperate species collected, *Coccinella septempunctata*, was only found at higher elevations with a more temperate climate.

We showed that psyllid abundance had a positive influence on the presence of all psyllophagous ladybird species, both adults and juveniles. Adults are mobile organisms that can disperse between different environments. Prey attracts and constitutes a stopping point for dispersing adults. Ladybirds lay their eggs in sites where they feed. Therefore, the factors that influence the distribution of larvae are likely to be similar to those affecting adult distribution [101,118]. Egg laying follows successful predation. Various factors influence the number of eggs laid and the duration of egg laying. For example, Honek (1980) identified that *C. septempunctata* starts laying when the aphid population reaches approximately one aphid per 300 cm² of leaf surface, a threshold that is consistent across different crops. Hemptinne and Dixon 1991 [32] reported that *A. bipunctata*, which prefers clusters of aphids, starts ovipositing at a higher minimum density of about two aphids per 150 cm²*.* In our case, adult psyllophagous ladybirds lay eggs on essential resources, which means the abundance of psyllids directly influences the presence of larvae.

Psyllid abundance positively influences the abundance of *E. laeviusculus* adults and juveniles, but not the other species. Due to its smaller size, *E. laeviusculus* can target psyllids at different stages of their development, often feeding on their small larvae or eggs. This has not been observed in larger species [119]. Compared to larger ladybirds, small aphid-eating ladybirds thrive even when aphid numbers are low. Smaller ladybirds may feed on colonies of aphids early in the aphid population increase, while larger species arrive later [31]. This can explain why *E. laeviusculus* responds better to psyllid density. Unlike generalist ladybirds, such as *E. laeviusculus*, specialist species, such as *O. v-nigrum*, only perform well on a few types of prey. This is a further demonstration that specialist ladybirds can persist at lower aphid densities than generalists [119,120]. In temperate continental environments, habitat generalists may have an advantage over specialists because, despite being less competitive, they can tolerate a wider range of environmental conditions [121,122]. Exotic species, including ladybirds, are also widely recognized as being more successful colonizers than native species [8,123], both in temperate continental [35,85] and insular [124] environments. In tropical island environments, ecological release often disfavors larger species, as competition with native species and resource limitations can hinder their establishment, as observed with *Harmonia axyridis* in the Azores [125]. This dynamic may benefit smaller species, allowing them to thrive in environments with fewer predators and abundant resources, thereby conserving energy [126]. Therefore, *Exochomus laeviusculus* has several natural advantages, namely, a generalist diet and a smaller size. In comparison, *Olla v-nigrum*, another island species, appears to be less competitive due to its own ecological release, despite being larger and more specialized.

Our results show that the landscape influenced the presence and abundance of adults of tropical and temperate psyllophagous ladybird species in a tropical island environment. Several authors have reported the influence of landscape diversity and fragmentation on insect communities [102,127,128], including ladybird species [33,129,130]. Lange et al. 2023 [128] also highlight that plant diversity encourages the establishment of natural grassland communities and stabilizes insect assemblages.

In addition, our results show that the landscape does not have the same influence on the three species that depend on psyllids. The presence and abundance of the generalist *E. laeviusculus* (both adults and juveniles) are influenced by the landscape at all levels (diversity, fragmentation and host plant covering). This is not the case for *C. septempunctata* and *O. v-nigrum*. Statistically, a more diverse landscape is favourable to generalist species because it offers a greater variety of prey [131,132], despite lower local abundance [133–135]. Landscape fragmentation hinders ladybird movement because in a fragmented landscape, encountering an unfavourable habitat is more likely [136]. The small size of *E. laeviusculus* may reduce its flying ability compared to *O. v-nigrum* and *C. septempunctata* [34]. The comparative size of the coccinellid and its prey is also a determining factor when it comes to feeding preference. An analysis of ladybirds’ prey indicates that the smaller the ladybird species, the smaller the prey and/or the greater the ladybird’s mobility [137]. This may explain why *E. laeviusculus* is more sensitive to landscape fragmentation than other species. More specialized ladybirds, such as *O. v-nigrum*, are less mobile because they can tolerate low prey densities [119], which means they are less influenced by the landscape. Lastly, according to the “predator satiation hypothesis” defined by Sweeney and Vannote [138] in 1982, an increase in host plant cover, correlated with prey availability at the landscape scale, dilutes *E. laeviusculus* populations.

## Conclusion

In conclusion, this study on ladybird species that interact with psyllid populations in Reunion island reveals unexpected species richness. We observed a mix of rare and predominant ladybird species, with a few common species. The community structure can be explained by the prey species associated with climatic parameters, such as monthly average temperature. It highlights the diversity and ecology of ladybird species, emphasizing the importance of both native and introduced species in the ecosystem. In the tropical island environment of Reunion Island, the findings underscore the influence of environmental factors like elevation, temperature and landscape on the ladybird species depending on their origin and life traits. This study highlights the unbalanced ratio between Hemiptera pests and predators, such as ladybirds, particularly at high elevations. More generally, the study helps improve our understanding of the role that ladybirds play in biological control [139] and ecosystem balance [140], offering insights for future conservation and management strategies in tropical environments.

## Supporting information

S1 Table Summary of landscape metrics for sampling sites, including site identification, fragmentation (number of patches), host plant covering (in square meters), and Simpson’s diversity index for landscape composition. (DOCX)

S2 Fig Spatial maps of sampling sites showing land-use categories within a 1-km radius, used for calculating landscape metrics such as fragmentation, host plant covering, and Simpson’s diversity index. (DOCX)
