# Supplementary material for: The impact of landscape and prey on psyllophagous ladybird communities in a tropical environment
Source: PLoS One. 2025 Apr 11;20(4):e0320898. doi: 10.1371/journal.pone.0320898 (PMC11991731; doi:10.1371/journal.pone.0320898)
Supplement: S1 Table — (DOCX) [file pone.0320898.s001.docx]

| *Site identification* | *Fragmentation* | *Host plant covering* | *Simpsons index* |
| --- | --- | --- | --- |
| *VOLH* | 50 | 106.25 | 0.23 |
| *VOLB* | 462 | 23.11 | 0.75 |
| *TH* | 929 | 41.34 | 0.73 |
| *MAIH* | 256 | 22.92 | 0.64 |
| *MAIB* | 277 | 170.15 | 0.50 |
| *ESP* | 508 | 87.50 | 0.56 |
| *ESL* | 1080 | 37.50 | 0.72 |
| *FES* | 458 | 100.00 | 0.26 |
| *MAK* | 802 | 1.00 | 0.65 |
| *BP* | 996 | 1.00 | 0.71 |
| *GA* | 755 | 118.75 | 0.62 |

**S1 Table.** **Summary of landscape metrics for sampling sites, including site identification, fragmentation (number of patches), host plant covering (in square meters), and Simpson's diversity index for landscape composition.**
