# Supplementary material for: The impact of landscape and prey on psyllophagous ladybird communities in a tropical environment
Source: PLoS One. 2025 Apr 11;20(4):e0320898. doi: 10.1371/journal.pone.0320898 (PMC11991731; doi:10.1371/journal.pone.0320898)
Supplement: S2 Fig — (DOCX) [file pone.0320898.s002.docx]

**S2 Fig.** **Spatial maps of sampling sites showing land-use categories within a 1-km radius, used for calculating landscape metrics such as fragmentation, host plant covering, and Simpson's diversity index.**

| *Acacia heterophylla* sites | |
| --- | --- |
| 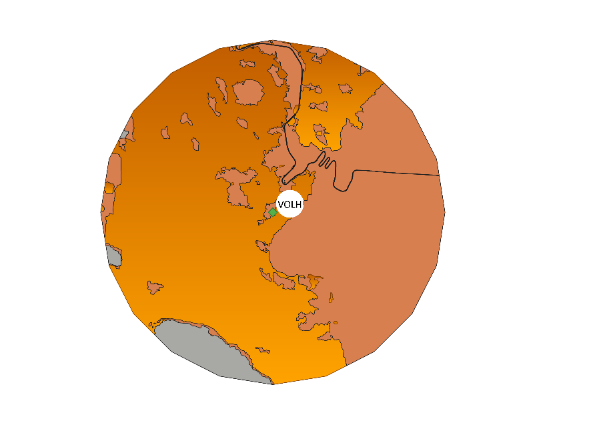 | 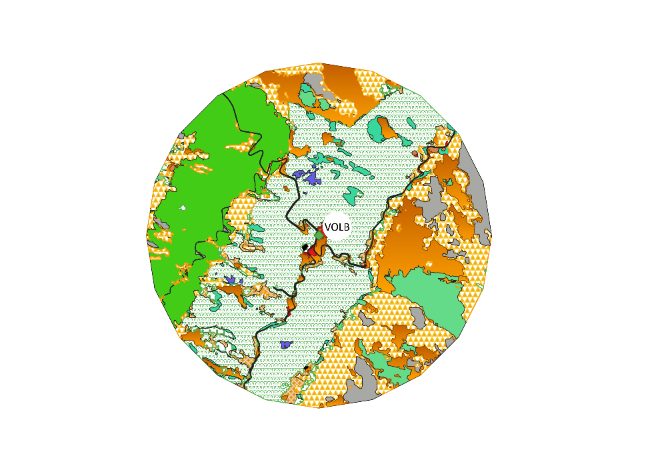 |
| 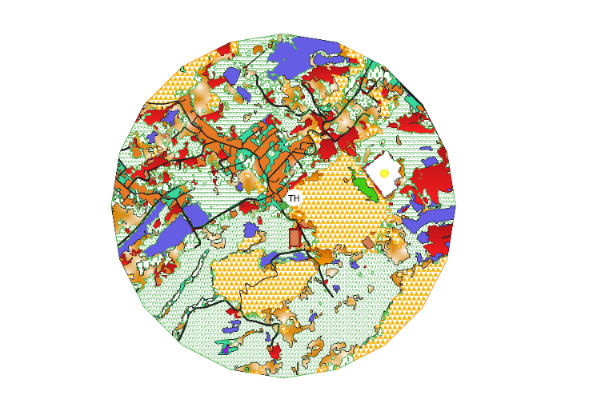 | 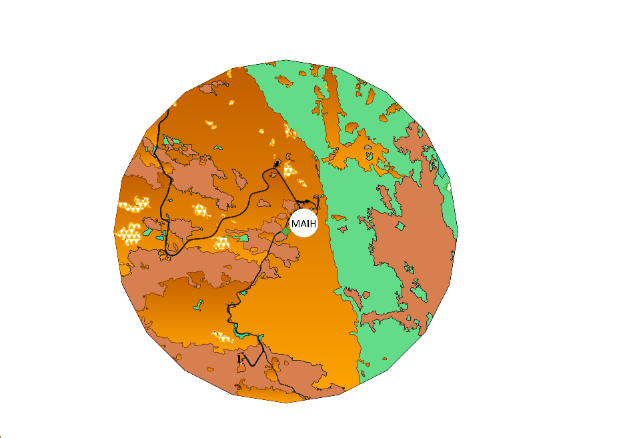 |
| 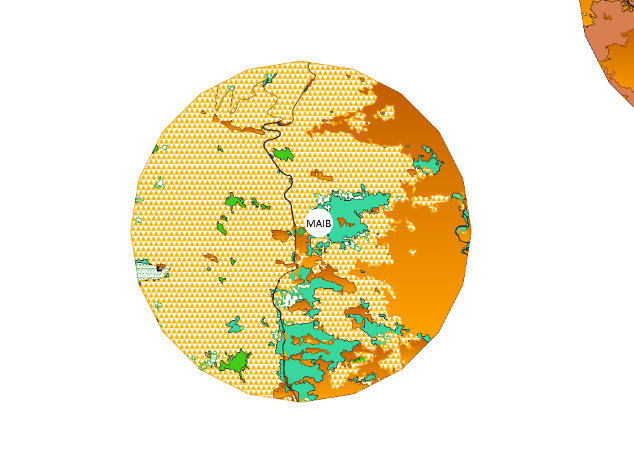 |  |
| *Leucaena leucocephala* sites | |
| 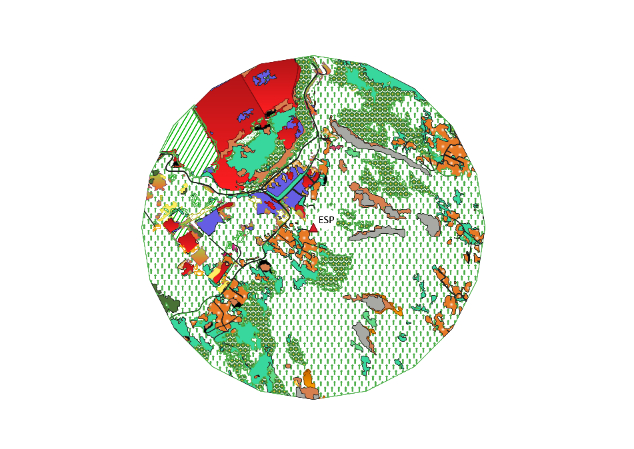 | *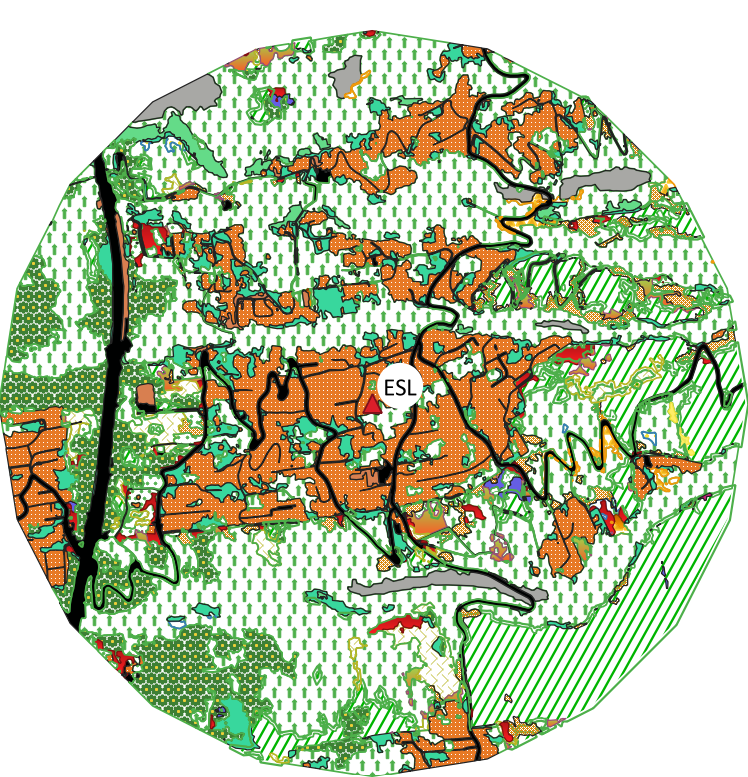* |
| 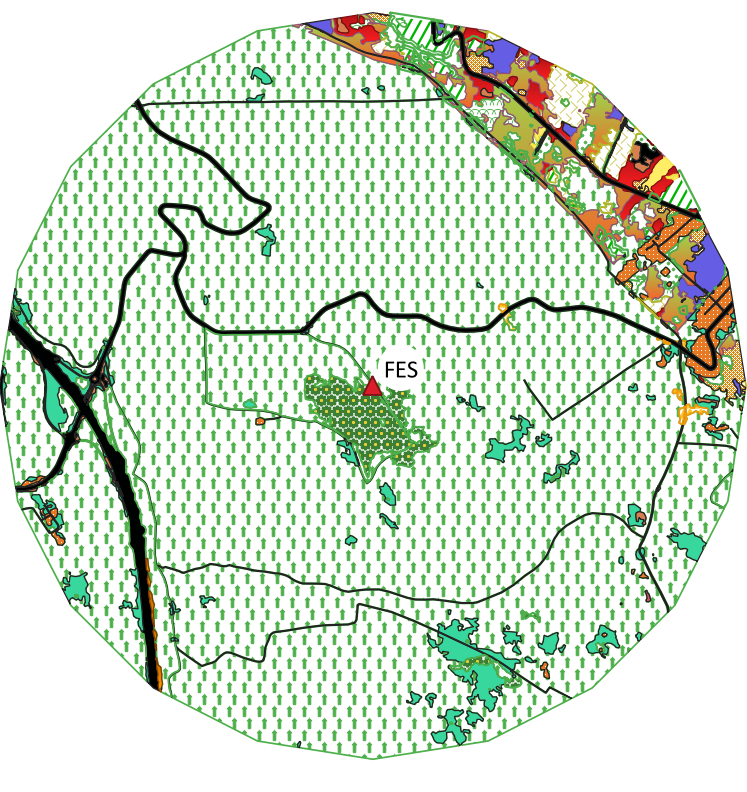 | 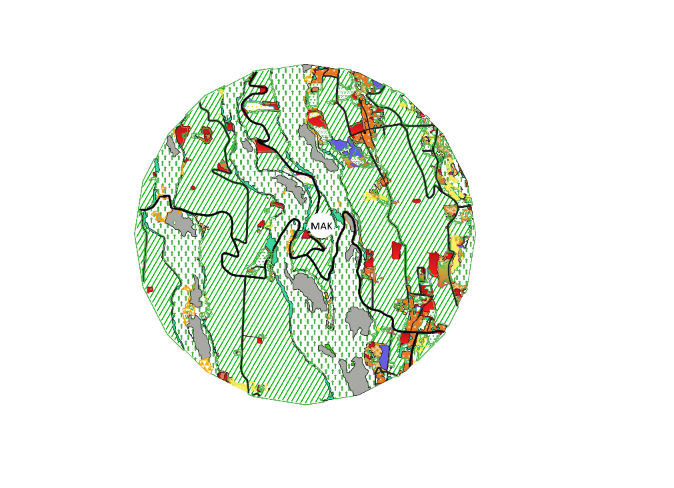 |
| 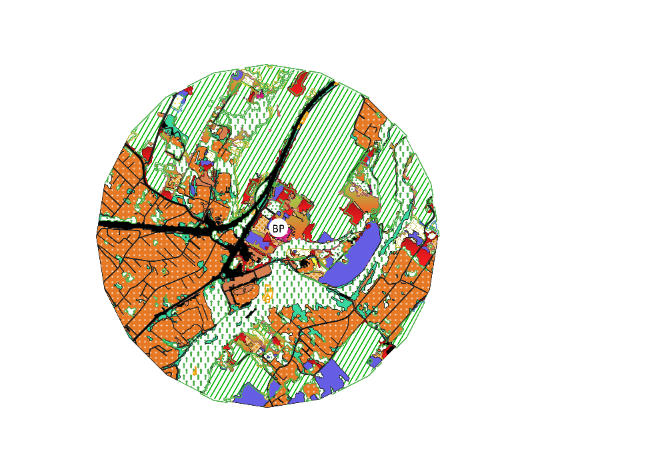 | 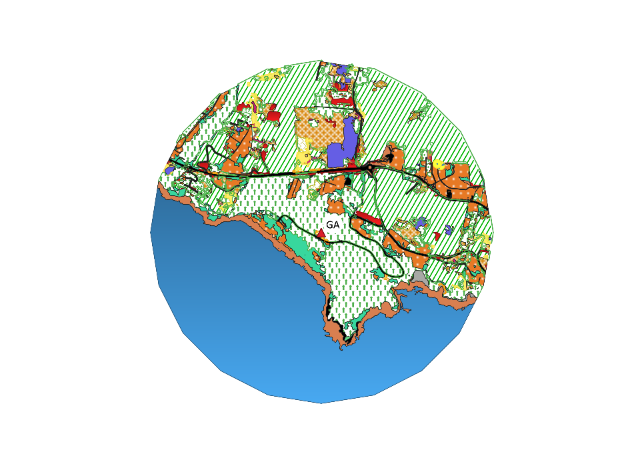 |
| Maps legend | |
| 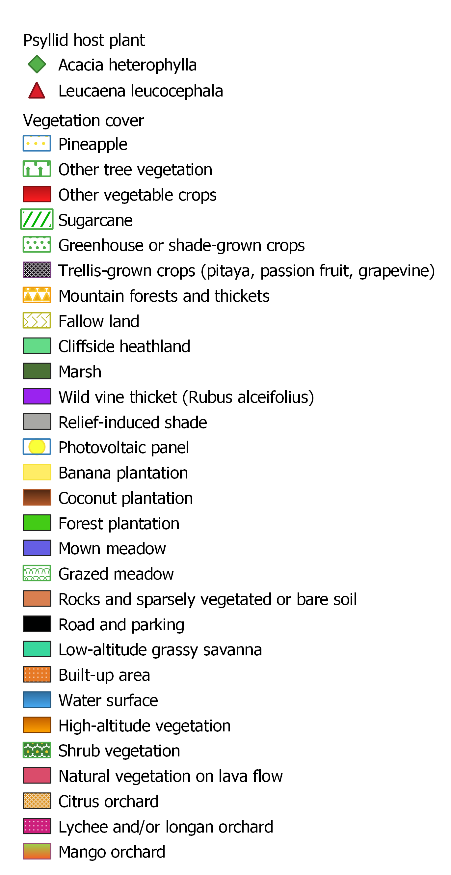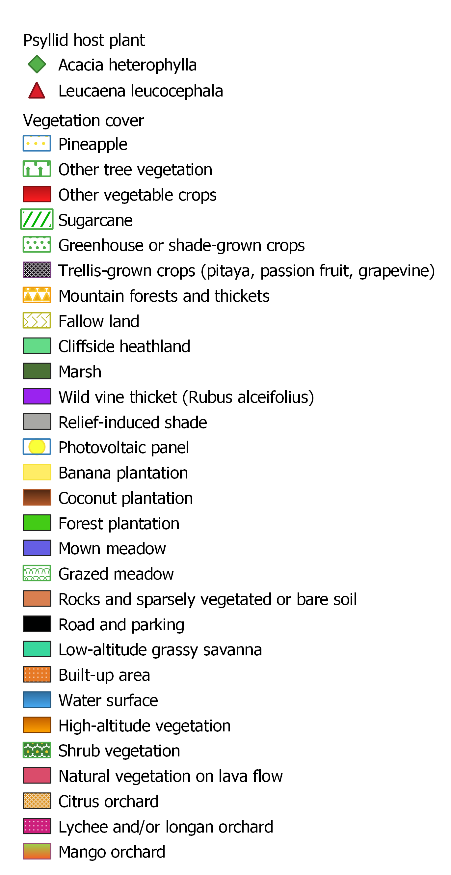 | |
